# Cierre de parafuga valvular con dispositivos *off - label*: un recurso valioso

**DOI:** 10.47487/apcyccv.v4i4.302

**Published:** 2023-12-27

**Authors:** Emilio Herrera, Alberto Navarro, Julián Vanegas, Juan C Ortiz

**Affiliations:** 1 Departamento de Medicina Interna, Universidad de Antioquia, Medellín, Colombia. Universidad de Antioquia Departamento de Medicina Interna Universidad de Antioquia Medellín Colombia; 2 Departamento de Cardiología, Hospital Alma Mater de Antioquia, Medellín, Colombia. Departamento de Cardiología Hospital Alma Mater de Antioquia Medellín Colombia

**Keywords:** Enfermedad Valvular del Corazón, Prótesis Valvular Cardiaca, Insuficiencia de la Válvula Aórtica, Heart Valve Disease, Cardiac Valve Prosthesis, Aortic Valve Insufficiency

## Abstract

Se presenta el caso de un paciente con parafuga valvular de prótesis mecánica en posición aórtica. Por intervención quirúrgica previa reciente se decide llevar a reparo percutáneo para disminuir riesgo perioperatorio, bajo indicación no aprobada (off label) usando un dispositivo para cierre de comunicación interventricular, exitoso y sin complicaciones en el seguimiento.

## Abstract (en)

We present the case of a patient with a paravalvular leak of mechanical prosthesis in aortic position. Due to a recent previous surgery, it was decided to perform a percutaneous repair to reduce perioperative risk, under an off-label indication using a device for ventricular septal defect closure, successful and without complications in the follow-up.

## Introducción

En países industrializados es más común la enfermedad valvular de origen degenerativo, y en aquellos en vía de desarrollo es más común la de origen reumático. En la mayoría de los casos en los que la enfermedad valvular cardiaca progresa a formas severas, requiere terapia de reemplazo valvular, casi siempre quirúrgico, de manera más reciente también percutáneo ^1^. En los Estados Unidos se implantan anualmente cerca de 60 000 válvulas cardiacas protésicas, de estas entre el 5 y hasta el 17% pueden desarrollar algún grado de insuficiencia paravalvular por múltiples mecanismos. La reintervención ha sido por muchos años la terapia estándar; sin embargo, no está exenta de riesgos y, en este sentido, el reparo por vía percutánea puede ser una alternativa razonable ^2^^,^^3^.

## Reporte de caso

Varón de 54 años con antecedente de reemplazo valvular aórtico con bioprótesis el año 2008 y recambio por prótesis mecánica Medtronic #25 en el 2022 debido a deterioro estructural. Por bloqueo aurículoventricular (AV) completo le fue implantado un marcapaso bicameral. En el curso del año 2022 tuvo trombosis de la prótesis tratada con fibrinólisis farmacológica de forma exitosa y luego anticoagulado con warfarina según *international normalized ratio* (INR).

Ingresa por cuadro clínico de 3 semanas consistente en disnea que progresa a mínimos esfuerzos y se asocia con edema de miembros inferiores y ortopnea. Examen clínico: peso 56 kg; talla 168 cm; presión arterial 107/57 mmHg; pulso 74/min; frecuencia respiratoria 16/min; saturando 95% al ambiente. Se destaca un *click* valvular y ausencia de soplo cardiaco. Presencia de edema de miembros inferiores simétrico grado I. No se evidencian otros hallazgos. Los exámenes iniciales revelan una bioquímica sanguínea sin hallazgos llamativos (sin evidencia de hemolisis) y un INR en metas. En electrocardiograma imagen de bloqueo completo de rama izquierda (BCRIHH) por estimulación del marcapaso.

Se realiza ecocardiograma transtorácico que destaca una fracción de eyección del ventrículo izquierdo (FEVI) del 30%, insuficiencia de la prótesis valvular aórtica grado severo, central, con velocidad máxima de 2,58 m/s, tiempo de aceleración de 67 m/s, relación de integrales de 0,48, dilatación de la raíz aortica en 41 mm y el resto de parámetros dentro de lo normal. 

**Figura 1:**
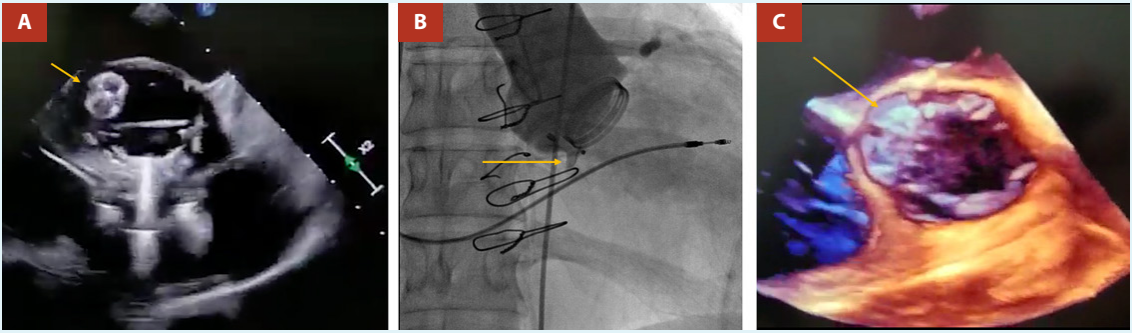
Imágenes pre procedimiento. **A)** Eco transtorácico, vista apical cinco cámaras, Doppler color evidenciando chorro de insuficiencia aórtica (flecha amarilla), **B)** cinefluoroscopía que muestra fuga del contraste con los discos protésicos cerrados, por fuera de la misma. **C)** Eco transesofágico, vista en eje corto, Doppler color evidenciando parafuga (flecha amarilla).

Se complementa con ecocardiograma transesofágico que muestra un ventrículo izquierdo dilatado con FEVI del 30% e hipocinesia difusa, aurículas de tamaño normal. TAPSE: 17 mm. Válvula mitral con dos *jets* de insuficiencia central leve. Prótesis mecánica en posición aórtica con anillo de 21 mm, sin *pannus*, apertura normal de los hemidiscos, gradiente máximo de 37 y medio de 18 mmHg; fuga paravalvular entre las 9 y las 12 del reloj hacia la continuidad mitroaórtica **(**Figura 1**)** con longitud de flujo de 19 mm, alcanzando el 25% de la circunferencia, y defecto anatómico de 9x6 mm. Vena contracta de 10 mm, tiempo de hemipresión de 190 ms y presión sistólica de arteria pulmonar (PSAP) de 49 mmHg. 

En el contexto clínico de falla cardiaca aguda, asociado al hallazgo ecocardiográfico de fuga paravalvular descrita, en un paciente con dos cirugías cardiacas previas, se decide llevar a reparo percutáneo del defecto.

**Figura 2:**
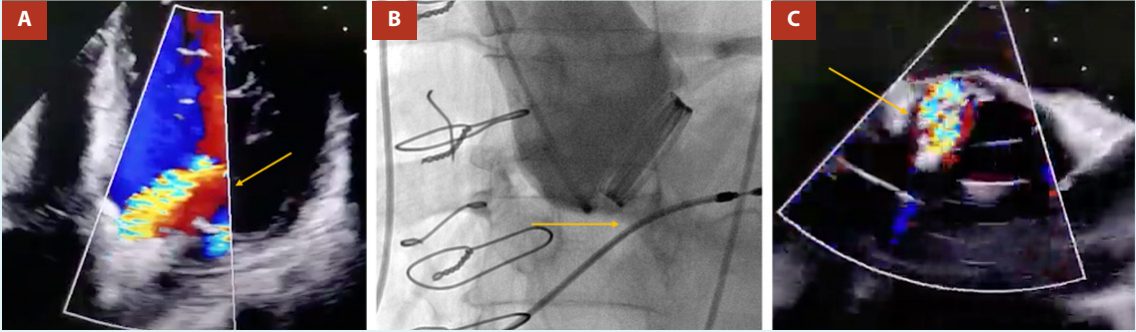
Imagen multimodal posprocedimiento. **A)** Eco transesofágico, vista en eje corto, **B)** Cinefluoroscopía y **C)** Eco en 3D de la válvula aórtica protésica, se observa el dispositivo tipo Amplatzer (flecha amarilla) en posición, en el sitio donde estaba la parafuga.

Se realiza reparo bajo anestesia cardiovascular, previo aortograma y con ecocardiograma transesofágico 3D. Se confirman los hallazgos. Se cruza el defecto usando guía hidrofílica al ventrículo izquierdo. Se avanza dispositivo para cierre de comunicación interventricular #8 (indicación *off label*). Se posiciona confirmado por ecocardiografía y fluoroscopía y se libera sin complicaciones **(**Figura 2**)**. La ecocardiografía en sala muestra disminución significativa del grado de insuficiencia. Se da alta 48 h después del procedimiento, asintomático. En seguimiento a 10 semanas buena condición clínica en clase funcional NYHA I.

## Discusión

Las fugas paravalvulares pueden presentarse después del reparo o reemplazo valvular (abierto o percutáneo). A pesar de que aun las fugas leves pueden tener un mal pronóstico a largo plazo, el tratamiento usualmente está indicado cuando causan compromiso ventricular (dilatación - disfunción), falla cardiaca, hemólisis y en el contexto de endocarditis. El paciente en discusión tenía compromiso severo de la función ventricular asociado a clínica de falla cardiaca. 

En el reemplazo valvular, la causa más común de parafuga suele ser la dehiscencia de las suturas. Esta puede ocurrir debido a factores relacionados con el paciente como tejido friable y muy calcificado o también factores externos como la presencia de endocarditis y la técnica de sutura ^(^^4^. La dehiscencia es más común en válvulas mecánicas que en bioprótesis y usualmente en posición mitral (cerca del 80% de los casos). Más de dos tercios ocurren en el primer año después del reparo valvular, igual que en el caso presentado. En estos pacientes se ha demostrado que la reintervención quirúrgica tiene una alta mortalidad comparada con el cierre percutáneo ^4^.

El diagnóstico suele ser desafiante. Cursan con la presencia de soplos leves que varían según la posición y trayectoria del chorro de insuficiencia. La evaluación por *Doppler* color puede estar comprometida por el artefacto que generan las válvulas mecánicas y las calcificaciones de los anillos valvulares. Con frecuencia es necesario el uso de ecocardiograma transesofágico para llegar al diagnóstico ^5^. En nuestro paciente se determinó el mecanismo y la severidad de la insuficiencia con ecocardiograma transesofágico, y se corroboraron los hallazgos con cinefluoroscopía. Para su evaluación se recomienda el uso de ecocardiograma 3D, ya que la forma del chorro suele ser irregular (generalmente media luna o rectangular - oblongado) al igual que su curso. La cuantificación de la severidad de la parafuga puede ser difícil; en el *Doppler* color se puede ver imagen en «manguera de jardín» en la cual pequeños orificios llenan todo el tracto de salida del ventrículo izquierdo, sin que exista una regurgitación severa o, por el contrario, la sombra acústica y demás artefactos de las válvulas mecánicas pueden subestimar la severidad del chorro regurgitante. El aortograma, la tomografía (de manera más reciente la tomografía fusionada con fluoroscopia), la resonancia cardiaca y la ecocardiografía intracardiaca son otras herramientas disponibles para abordar casos que generen duda ^5^^,^^6^.

Actualmente no se dispone de una oferta importante en términos de dispositivos creados exclusivamente para dicho fin, la mayoría son de uso *off label*. Los más usados son los de la familia Amplatzer para cierre de defectos septales. Disponibles en varios tamaños, generalmente se eligen un poco más grandes que el defecto para garantizar su oclusión completa, cuidando que no supere el borde interno del marco valvular e interfiera con el mecanismo de apertura y cierre de la prótesis, puesto que esta complicación puede ser muy grave ^7^. Una vez posicionado el dispositivo de cierre, se confirma con imágenes y se evalúa el grado de regurgitación residual. Lo cual se hizo en nuestro caso.

Se recomienda el seguimiento con imágenes, principalmente a los 6 meses, para evaluar la estabilidad del dispositivo, la efectividad del cierre y si hay fuga residual. La mejoría clínica se ve en un 67 a 76% de los pacientes ^8^. Las complicaciones son potencialmente graves e incluyen embolización del dispositivo, interferencia con los discos de la prótesis valvular, tromboembolismo o embolismo aéreo, taponamiento cardiaco y sangrado. Otras complicaciones son menos frecuentes como la disección de aorta y la obstrucción de los *ostium* coronarios.

A pesar de que existen pocos datos del pronóstico a largo plazo después del cierre, la fuga residual parece no ser buena predictora de sobrevida, pero si puede generar síntomas. Por el contrario, la hemólisis sí parece ser un marcador de mortalidad. En este sentido, cuando el objetivo es la mejoría de falla cardiaca, cualquier disminución en el volumen regurgitante es favorable para el paciente. En cambio, si la indicación de cierre es hemólisis, el objetivo debe ser el cierre completo del defecto. Algunas series han reportado sobrevida cercana al 86% a 18 meses y 64% a 5 años ^9^^,^^10^.

En conclusión, el caso presentado resalta la importancia del cierre percutáneo de estos tipos de defectos como alternativa al manejo quirúrgico que tiene un mayor riesgo para el paciente. El uso de dispositivos de manera *off label* es una herramienta que puede ayudar en el abordaje de estos casos que en principio son desafiantes.

## Fuente de financiamiento:

Autofinanciado
